# Rethinking Plasticity in Sponges

**DOI:** 10.1002/jez.b.70035

**Published:** 2026-08-03

**Authors:** Laura Oliveira, Emilio Lanna

**Affiliations:** ^1^ Universidade Federal da Bahia, Instituto de Biologia, Laboratório de Biologia Evolutiva e Desenvolvimento, LABED ‐ UFBA Salvador Brazil; ^2^ Departamento de Biologia Faculdade de Filosofia, Ciências e Letras de Ribeirão Preto Brazil; ^3^ National Institute of Science and Technology in Interdisciplinary and Transdisciplinary Studies in Ecology and Evolution (INCT IN‐TREE) Salvador Brazil

**Keywords:** adaptive, cellular plasticity, genotype versus environment interactions, levels of plasticity, morphological plasticity, population‐level

## Abstract

What lies behind the extraordinary phenotypic plasticity of sponges? We used this question to conduct a systematic review of how “phenotypic plasticity” is approached in the literature regarding the phylum Porifera. First, we gathered information on what the literature says about plasticity in sponges. Next, we analyzed its usage contexts, distinguishing between plasticity as a general feature versus a specific process linked to environmental triggers or genetic variation. Thus, we critique the overuse of synonymous terms that fail to capture the concept's complexity and advocate for greater precision to advance research. Finally, we advocate for precise and consistent terminology as an essential requirement for advancing research on sponge phenotypic plasticity. Phenotypic plasticity in sponges is a multifaceted phenomenon. We focused on the rationale behind the term's use and its underlying biological assumptions, proposing a categorization into three levels: (1) intraindividual/cellular plasticity, regarding modifications in a single organism's structures; (2) individual plasticity, involving whole‐organism adaptation; and (3) population‐level plasticity, where variability occurs among individuals of the same species. This framework distinguishes between changes within a single organism and broader variation patterns, highlighting scale‐specific biological constraints. Recognizing sponges as highly plastic organisms was only the beginning. We advanced our comprehension by addressing key evolutionary questions and knowledge gaps that remain essential to guide future research on the molecular, ecological, and evolutionary implications of plasticity in this phylum.

## Graphical abstract

This systematic review critiques the conceptual confusion caused by the indiscriminate use of “plasticity” in Porifera literature, revealing that researchers often overlook genetic variability and fail to distinguish between real‐time environmental responses and natural selection. To address these gaps, the study proposes a standardized framework categorizing plasticity into intraindividual/cellular, individual, and population levels, effectively separating specific biological responses from broader evolutionary patterns.

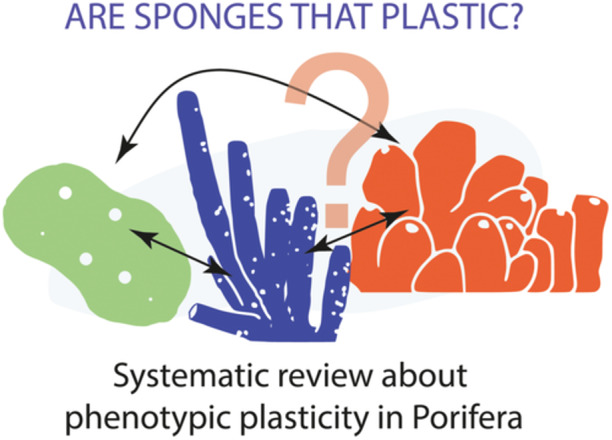

## 1 Introduction

A key challenge in biology has been understanding how life adjusts to different environmental conditions. Evolutionary biology has traditionally approached this question through the adaptive evolution model: organisms adjust through adaptations. In this model, the environment has been placed primarily as a selective agent, driving changes through fundamental evolutionary processes such as natural selection, mutation, migration, and genetic drift, leading to changes in heritable traits over time. However, this perspective has historically overlooked more dynamic, reciprocal mechanisms, such as phenotypic plasticity.

Phenotypic plasticity is fundamentally simple and well‐defined. We therefore do not aim to provide an exhaustive review, as others have already discussed these concepts extensively (see, for instance, Box 1 in Whitman and Agrawal 2009). The “commonest” definition is the ability of a single genotype to produce two or more phenotypes in response to changes in the environment (West‐Eberhard 1989; Pigliucci 2001). As a fundamental property of the phenotype, plasticity unites perhaps all of biology (Bradshaw 1965; Pigliucci 2001; West‐Eberhard 2003). It spans all organizational levels (e.g., genes, individuals, and populations), serving as an “umbrella” term for diverse biological responses.

In recent decades, phenotypic plasticity has gained significant attention in research as a potential pivotal evolutionary mechanism (West‐Eberhard 1989). It can promote adaptive change and diversification when: (i) selection refines plasticity into a new adaptive phenotype (plasticity‐first evolution), or (ii) plasticity enables survival in heterogeneous environments. Conversely, plasticity can be non‐adaptive if it: (i) impedes adaptive change by “hiding” traits from selection, slowing evolution, or (ii) renders local adaptation redundant with no fitness effect (West‐Eberhard 1989; Pigliucci 2001; Whitman and Agrawal 2009). A crucial dimension of plasticity is developmental plasticity, which shapes individual phenotypes and influences evolutionary trajectories by altering trait expression and integration during critical life stages (West‐Eberhard 2003). In this framework, plasticity in pre‐embryonic development reveals existing genetic variations that might otherwise remain “hidden” and unavailable for selection (Gilbert et al. 2015). This ability in developmental processes explains the environment's role as a variation inducer, illustrating how organisms adapt to changing conditions.

The pervasiveness of phenotypic plasticity varies across lineages, but, among animals, Porifera (sponges) stands out. Sponges are broadly known as sessile, aquatic, and benthic animals with a long history of life on Earth. The phylum comprises four extant classes: Calcarea, Homoscleromorpha, Demospongiae, and Hexactinellida. As the likely sister group to all metazoans, sponges offer insights into early animal evolution (Redmond and McLysaght 2021), sharing molecular and developmental similarities with the rest of Metazoa. Morphologically, they exhibit vast functional diversity and unique anatomy (Leys and Hill 2012). Except for carnivorous species (Cladorhizidae, Demospongiae), most sponges possess an aquiferous system that functions as a pumping system to optimize filtration (Cavalcanti and Klautau 2011). This essential network is remodeled throughout life to meet environmental or physiological demands, challenging conventional notions of animal developmental rigidity and environmental influence (Colgren and Nichols 2020).

Sponges strike a different balance between patterning and plasticity, making these animals very important for understanding the environmental role in inducing variation and developmental evolution. They may even exhibit high levels of morphological variation within the same species due to strong environmental influences, leading to uncertainty about what determines their morphology (Bell 2007; Lanna et al. 2017; Schönberg 2021; Cajado and Lanna 2021; Wulff 2023). Their external shapes are typically influenced by external factors such as hydrodynamic conditions, substrate type, sedimentation, and the presence of symbionts (Wulff 2006; Bell et al. 2015; Schönberg 2021). However, some species do have characteristic shapes, possibly indicating the existence of phylogenetic constraints (Schönberg 2021). There are two main ways to explain such variation. First, genetic differentiation (polymorphism), where different genotypes produce different phenotypes. Second, high phenotypic plasticity, in which the environment induces changes. These scenarios are not mutually exclusive and may operate simultaneously to generate this variation (Foster 1979; Amaral 1994; Todd 2008).

Conflict arises when sponge phenotypic plasticity is used synonymously with intraspecific variation, which may instead stem from genetic differentiation (Wulff 2023). This conceptual confusion has led to an unrealistic dimension of plasticity, making it a generalized attribute within the phylum. Distinguishing what is plasticity and what is not becomes even more complex, as few studies have investigated the true plastic capacity of these animals. This major gap in knowledge of sponge biology raises open questions about the biology and evolution of the phylum, e.g., the origin of variation, the establishment of connections between genotypes and phenotypes, and responsiveness to the environment, including adaptability to environmental changes.

Our investigation aims to examine the use of the term “phenotypic plasticity” in the literature related to the phylum Porifera to better understand its evolutionary and ecological implications in sponges.

## 2 Materials and Methods

**Figure 1:**
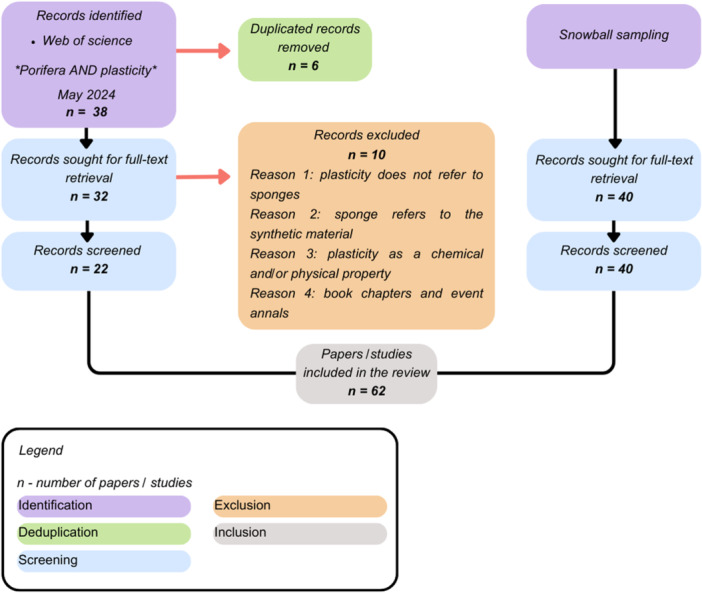
Research flowchart with PRISMA model, showing the research steps, number of articles included, excluded, and exclusion criteria to investigate the use of phenotypic plasticity in the literature dealing with Porifera.

We conducted a systematic review following the PRISMA (Preferred Reporting Items for Systematic Reviews and Meta‐Analyses) protocol (Moher et al. 2015) to synthesize relevant articles, using a flow diagram to illustrate the sampling strategy (Figure 1). We performed a search in May 2024 to find publications on phenotypic plasticity in sponges on the Web of Science database. We found a total of 38 studies applying the terms: *Porifera AND plasticity*. The second step was manually removing duplicates (4 studies), using Mendeley Reference Manager (2 studies), and removing inaccessible publications (4 studies), leaving 28 records. We screened titles, abstracts, and keywords, excluding 6 records where: (i) plasticity did not refer to sponges, (ii) “sponge” meant synthetic material, (iii) plasticity was a chemical/physical property, or (iv) the record was a book chapter/event annals. This approach left 22 records for full‐text retrieval. We complemented our review with snowball sampling, searching for “plasticity” and “Porifera” within the initial publications’ references. This identified 40 additional papers, bringing the total to 62 publications. Note that some publications were inevitably omitted, an intrinsic caveat of systematic reviews.

We categorized the 62 publications (Table 1) using the authors’ original terminology to provide an overview of sponge plasticity. Articles were grouped by whether they used “phenotypic plasticity” (or its synonyms) as (a) a descriptive feature or (b) discussed it as a central phenomenon within their hypotheses or findings.

**Table 1:** Summary of the terminology used in the literature about the phenotypic plasticity of sponges and main terms used as synonyms for plasticity.

	Source
Specific terminology for phenotypic plasticity found in Porifera articles	
Acclimation	Palumbi (1986); Carballo et al. (2006); Enríquez et al. (2009)
Acclimatization	Trani et al. (2021)
Developmental plasticity	Colgren and Nichols (2020)
Phenotypic plasticity	Bavestrello and Sarà (1992); Barbieri et al. (1995); Miller et al. (2001); Pronzato et al. (1998); Hill and Hill (2002); Carballo et al. (2006); Enríquez et al. (2009); López‐Legentil et al. (2010); Dailianis et al. (2011); Andreakis et al. (2012); Freckelton et al. (2012); Guardiola et al. (2016); Manconi and Pronzato (2016); Morley et al. (2016); Slattery et al. (2016); Galitz et al. (2018); Castillo‐Páez et al. (2021); Gost et al. (2023); Castillo‐Páez et al. (2024); Pulido Mantas et al. (2024)
Plasticity	Pronzato and Manconi (1994); Gaino et al. (1995); Koziol et al. (1998); Müller (1998); Klautau and Valentine (2003); Cavalcanti et al. (2007); Blanquer and Uriz (2007); Gaino et al. (2009); Nakayama et al. (2015); Padua et al. (2016); Lavrov and Kosevich (2016); Finoshin et al. (2020); Vicente et al. (2019); Sacristán‐Soriano et al. (2020); Adameyko et al. (2021); Colgren and Nichols (2022); Kravchuk et al. (2023); Pulido Mantas et al. (2024)
Main terms used as synonyms for plasticity in Porifera articles	
Anatomical plasticity, body plan plasticity; morphological plasticity, morphologically plastic, growth‐form plasticity; plasticity of body plan; plasticity of morphological characters, structural plasticity, skeletal plasticity; plastic morphological features; and macromorphological plasticity	Gaino et al. (1995); Pronzato et al. (1998); Hill and Hill (2002); Mercurio et al. (2006); Meroz‐Fine et al. (2005); Carballo et al. (2006); Klautau and Valentine (2003); Enríquez et al. (2009); López‐Legentil et al. (2010); Andreakis et al. (2012); Freckelton et al. (2012); Ereskovsky et al. (2013a); Knapp et al. (2015); Lavrov and Kosevich (2016); Manconi and Pronzato (2016); Padua et al. (2016); Willenz et al. (2016); Shaffer et al. (2018); Lopes et al. (2018); Vicente et al. (2019); Finoshin et al. (2020); Gökalp et al. (2020); Castillo‑Páez et al. 2021; Wulff (2023); Pulido Mantas et al. (2024)
Morphogenetic plasticity	Ereskovsky et al. (2015)
Intraspecific plasticity, intraspecific morphological plasticity; intra‐species plasticity; plasticity of ecological characters; and environmental plasticity	Blanquer and Uriz (2007); Valderrama et al. (2009); Boury‐Esnault et al. (2013); Shaffer et al. (2018); Sacristán‐Soriano et al. (2020); Castillo‑Páez et al. 2021; Trani et al. (2021); Wulff (2023)
Adaptational plasticity	Mukherjee et al. (2016)
Organizational plasticity and morphofunctional plasticity	Gaino et al. (1995); Nickel (2006); Knapp et al. (2015); Stocchino et al. (2021)
Physiological plasticity; plasticity of seasonal responses; reproductive plasticity; plasticity of reproductive traits; tissue plasticity; plasticity of tissue organization; and sexual phenotypic plasticity	Meroz‐Fine et al. (2005); Gilis et al. (2011); Fassini et al. (2012); Morley et al. (2016); Idan et al. (2020)
Sponge plasticity	Gaino et al. (1995); Finoshin et al. (2020)
Cell plasticity, cellular plasticity; differential plasticity; plasticity of cell differentiation; plasticity in the determination of cell lineages; and plasticity of cell differentiations	Koziol et al. (1998); Müller and Müller (2003); Lavrov and Kosevich (2016); Funayama (2018); Finoshin et al. (2020); Kravchuk et al. (2023)

We continued the analysis with the later category of publications (i.e., those discussing plasticity as a present phenomenon in the hypothesis and/or findings, excluding review studies), classifying responses as: (a) morphological [internal (tissues, skeleton, spicules) or external (color, shape, growth form, oscula)], (b) physiological (filtration, biochemistry, cellular activity, reproduction, growth), (c) molecular (gene and protein expression), or (d) developmental (pre‐adult changes). Some publications overlap by addressing multiple response types, such as morphology and physiology or molecular and developmental simultaneously. Categories such as morphological, molecular, or developmental simply reflect the study's focus and the traits quantified. Consequently, we did not categorize specific subtypes (e.g., developmental plasticity, polyphenism, phenotypic flexibility, life‐cycle staging).

Still with publications of the second category, we identified the external factors that triggered the phenotypically plastic responses (depth, tides, seasonality, commensalism, symbiosis, predation, hydrodynamic conditions, alkalinity, sedimentation, substrate, temperature, irradiation, dissolved organic matter, pH, oxygen level, and iron concentration) in the articles that presented these variables in the methodology. As depth, tides, and seasonality are subjective environmental factors, we only kept them for articles that did not specify the exact variables involved. For each of the 62 total studies, we characterized the taxa (class and species) and life stage (gemmule/bud, embryo/larval, primmorph, or adult) (Table 2). Some studies overlap by examining the same species across different stages.

**Table 2:** Summary of the data about the studies on the phenotypic plasticity in sponges, indicating the investigated class, species, and life stages. (*Life stage refers to the stage of the life cycle in which the work was carried out: Embryo/larva, gemmule/bud, primmorph, or adult). Species are organized per class and listed in alphabetical order.

Class	Species	Life stage*	References
Calcarea	*Clathrina aurea* Solé‐Cava, Klautau, Boury‐Esnault, Borojevic and Thorpe, 1991	Adult	Padua et al. (2016)
Calcarea	*Clathrina lutea* Azevedo, Padua, Moraes, Rossi, Muricy and Klautau, 2017	Adult	Lopes et al. (2018)
Calcarea	*Clathrina smaragda* Lopes et al. (2018)	Adult	Lopes et al. (2018)
Calcarea	*Clathrina* sp.	Adult	Klautau and Valentine (2003)
Calcarea	*Ernstia rocasensis* Azevedo, Padua, Moraes, Rossi, Muricy and Klautau, 2017	Adult	Lopes et al. (2018)
Calcarea	*Leucetta floridana* (Haeckel, 1872)	Adult	Valderrama et al. (2009)
Calcarea	*Paraleucilla magna* Klautau, Monteiro and Borojevic, 2004	Adult	Guardiola et al. (2016) Lanna et al. (2017)
Calcarea	*Petrobiona massiliana* Vacelet and Lévi, 1958	Adult	Gilis et al. (2011)
Homoscleromorpha	*Oscarella lobularis* (Schmidt, 1862)	Adult	Ereskovsky et al. (2013a) Ereskovsky et al. (2015)
Homoscleromorpha	*Oscarella tuberculata* (Schmidt, 1868)	Adult	Ereskovsky et al. (2013a)
Homoscleromorpha	*Plakortis angulospiculatus* (Carter, 1879)	Adult	Slattery et al. (2016)
Demospongiae	*Anthosigmella varians* (Duchassaing and Michelotti, 1864)	Adult	Hill and Hill (2002)
Demospongiae	*Aplysina aerophoba* (Nardo, 1833)	Adult	Wilkinson and Vacelet (1979)
Demospongiae	*Aplysina cauliformis* (Carter, 1882)	Adult	Wulff (2023)
Demospongiae	*Aplysina cavernicola* (Vacelet, 1959)	Adult	Wilkinson and Vacelet (1979)
Demospongiae	*Aplysina fulva* (Pallas, 1766)	Adult	Wulff (2023)
Demospongiae	*Axinella antarctica* (Koltun, 1964)	Adult	Morley et al. (2016)
Demospongiae	*Axinella corrugata* (George and Wilson, 1919)	Adult	Blanquer and Uriz (2007)
Demospongiae	*Axinella polypoides* Schmidt, 1862	Adult	Idan et al. (2020)
Demospongiae	*Callyspongia (Callyspongia) fallax* Duchassaing and Michelotti, 1864	Adult	López‐Legentil et al. (2010)
Demospongiae	*Callyspongia (Cladochalina) plicifera* (Lamarck, 1814)	Adult	López‐Legentil et al. (2010)
Demospongiae	*Callyspongia (Cladochalina) aculeata* (Linnaeus, 1759)	Adult	Trussell et al. (2006) López‐Legentil et al. (2010)
Demospongiae	*Calyx arcuarius* (Topsent, 1913)	Adult	Morley et al. (2016)
Demospongiae	*Cervicornia cuspidifera* (Lamarck, 1815)	Adult	Sacristán‐Soriano et al. (2020)
Demospongiae	*Chondrosia reniformis* Nardo, 1847	Adult	Wilkinson and Vacelet (1979) Fassini et al. (2012) Idan et al. (2020) Gökalp et al. (2020)
Demospongiae	*Chondrilla nucula* Schmidt, 1862	Adult	Wilkinson and Vacelet (1979)
Demospongiae	*Chondrilla* aff. *nucula*	Adult	Cavalcanti et al. (2007)
Demospongiae	*Clathria (Axociella) nidificata* (Kirkpatrick, 1907)	Adult	Morley et al. (2016)
Demospongiae	*Cliona delitrix* Pang, 1973	Adult	Sacristán‐Soriano et al. (2020)
Demospongiae	*Cliona nigricans* (Schmidt, 1862)	Adult	Barbieri et al. (1995)
Demospongiae	*Cliona tumula* Friday, Poppell and Hill, 2013	Adult	Sacristán‐Soriano et al. (2020)
Demospongiae	*Cliona varians* (Duchassaing and Michelotti, 1864)	Adult	Sacristán‐Soriano et al. (2020)
Demospongiae	*Cliona viridis* (Schmidt, 1862)	Adult	Barbieri et al. (1995)
Demospongiae	*Dendrilla antarctica* Topsent, 1905	Adult	Morley et al. (2016)
Demospongiae	*Dictyonella incisa* (Schmidt, 1880)	Adult	Blanquer and Uriz (2007)
Demospongiae	*Dictyonella* sp.	Adult	Blanquer and Uriz (2007)
Demospongiae	*Ephydatia fluviatilis* (Linnaeus, 1759)	Gemmule/bud	Nakayama et al. (2015) Gost et al. (2023)
Demospongiae	*Ephydatia muelleri* (Lieberkühn, 1856)	Adult	Colgren and Nichols (2022)
Demospongiae	*Eunapius carteri* (Bowerbank, 1863)	Adult	Mukherjee et al. (2016)
Demospongiae	*Geodia cydonium* (Linnaeus, 1767)	Adult	Koziol et al. (1998) Müller and Müller (2003) Mercurio et al. (2006)
Demospongiae	*Halichondria (Halichondria) panicea* (Pallas, 1766)	Embryo/larva Primmorph	Palumbi (1986) Lavrov and Kosevich (2016) Finoshin et al. (2020) Adameyko et al. (2021)
Demospongiae	*Haliclona (Reniera) aquaeductus* (Schmidt, 1862)	Primmorph	Lavrov and Kosevich (2016)
Demospongiae	*Haliclona (Soestella) caerulea* (Hechtel, 1965)	Adult	Enríquez et al. (2009) Carballo et al. (2006)
Demospongiae	*Halisarca dujardinii* Johnston, 1842	Primmorph Adult	Lavrov and Kosevich (2016) Kravchuk et al. (2023)
Demospongiae	*Halisarca magellanica* Topsent, 1901	Adult	Willenz et al. (2016)
Demospongiae	*Hemigellius* sp.	Adult	Morley et al. (2016)
Demospongiae	*Homaxinella balfourensis* (Ridley and Dendy, 1886)	Adult	Morley et al. (2016)
Demospongiae	*Ianthella basta* (Pallas, 1766)	Adult	Andreakis et al. (2012) Freckelton et al. (2012)
Demospongiae	*Ircinia variabilis* (Schmidt, 1862)	Adult	Wilkinson and Vacelet (1979)
Demospongiae	*Isodictya kerguelenensis* (Ridley and Dendy, 1886)	Adult	Morley et al. (2016)
Demospongiae	*Latrunculia* sp.	Adult	Miller et al. (2001)
Demospongiae	*Lendenfeldia chondrodes* (de Laubenfels, 1954)	Adult	Galitz et al. (2018)
Demospongiae	*Mycale (Oxymycale) acerata* Kirkpatrick, 1907	Adult	Morley et al. (2016)
Demospongiae	*Mycale (Carmia) cecilia* de Laubenfels, 1936	Adult	Castillo‐Páez et al. (2021) Castillo‐Páez et al. (2024)
Demospongiae	*Neopetrosia* spp.	Adult	Vicente et al. (2019)
Demospongiae	*Petrosia (Petrosia) ficiformis* (Poiret, 1789)	Adult	Bavestrello and Sarà (1992)
Demospongiae	*Phorbas* sp.	Adult	Morley et al. (2016)
Demospongiae	*Sarcotragus foetidus* Schmidt, 1862	Adult	Pulido Mantas et al. (2024)
Demospongiae	*Sarcotragus spinosulus* Schmidt, 1862	Adult	Trani et al. (2021) Stocchino et al. (2021)
Demospongiae	*Scopalina blanensis* Blanquer et al. (2008)	Adult	Blanquer et al. (2008)
Demospongiae	*Scopalina lophyropoda* Schmidt, 1862	Adult	Blanquer and Uriz (2007) Blanquer et al. (2008)
Demospongiae	*Scopalina ruetzleri* (Wiedenmayer, 1977)	Adult	Blanquer and Uriz (2007)
Demospongiae	*Sphaerotylus antarcticus* Kirkpatrick, 1907	Adult	Morley et al. (2016)
Demospongiae	*Spheciospongia vesparium* (Lamarck, 1815)	Adult	Sacristán‐Soriano et al. (2020)
Demospongiae	*Spongia (Spongia) agaricina* Pallas, 1766	Adult	Pronzato et al. (1998)
Demospongiae	*Spongia (Spongia) officinalis* Linnaeus, 1759	Adult	Pronzato et al. (1998) Dailianis et al. (2011)
Demospongiae	*Spongia (Spongia) virgultosa* (Schmidt, 1868)	Adult	Pronzato et al. (1998)
Demospongiae	*Suberites domuncula* (Olivi, 1792)	AdultPrimmorph	Koziol et al. (1998) Müller and Müller (2003)
Demospongiae	*Suberites* sp.	Adult	Morley et al. (2016)
Demospongiae	*Tethya bergquistae* Hooper in Hooper and Wiedenmayer, 1994	Adult	Shaffer et al. (2018)
Demospongiae	*Tethya burtoni* Sarà and Sarà, 2004	Adult	Shaffer et al. (2018)
Demospongiae	*Tethya wilhelma* Sarà, Sarà, Nickel and Brümmer, 2001	Adult	Nickel (2006)
Demospongiae	*Tethya seychellensis* (Wright, 1881)	Adult	Gaino et al. (2009)
Demospongiae	*Tetilla* sp.	Adult	Meroz‐Fine et al. (2005)

Given the dual nature of sponge plasticity (i.e., variation between individuals probably based in genetic diversity; and plasticity of individuals triggered by changes in the external environment) and the different levels of plasticity (e.g., genes, individuals and populations), we categorized the publications of the second category into three levels of plastic response: (I) individual level (i.e., environmentally induced changes in a single genotype), (II) population level (i.e., induced changes between potentially different genotypes)) and (III) intraindividual/cellular level (i.e., differentiation among cells and tissues, with or without environmental influence).

## 3 Results and Discussion

### 3.1 Systematic Review: Quali‐Quantitative Analysis of Phenotypic Plasticity in Sponges

We found 62 studies based on the criteria established here, with the oldest record being Wilkinson and Vacelet (1979), and the most recent being Castillo‐Páez et al. (2024) and Pulido Mantas et al. (2024). Our systematic review identified a higher preference for using synonymous terms of plasticity rather than plasticity‐specific terminology in the literature about sponges. As an "umbrella" term for related concepts, plasticity is frequently, and often unconditionally, interchanged with its synonyms. We also noticed the exaggerated use of synonyms related to sponge morphology, such as "anatomical plasticity", "body plan plasticity", "morphological plasticity", "growth‐form plasticity", "plasticity of morphological characters", "structural plasticity", "skeletal plasticity", "plastic morphological features", and "macromorphological plasticity" (Table 1). This tendency reflects a distinct feature of the phylum: sponges have an alleged flexible morphology (Van Soest et al. 2012). While alternative terms may highlight unique forms, authors must clarify their interpretations to avoid confusion (Forsman 2015), as relying on synonyms can overlook nuances and create ambiguities that hamper comparative research.

The overuse of synonyms creates three main issues: (1) retrieval difficulties, as inconsistent terminology hinders literature searches and meta‐analyses; (2) knowledge fragmentation, which prevents a unified understanding across ecological contexts; and (3) reduced accuracy, where vague terms obscure biological mechanisms. In sponge research, this has caused long‐standing confusion; for example, genetic variability is often mistaken for plasticity and vice versa (Wulff 2023). This lack of "identity" in Porifera studies, driven by the indiscriminate use of synonyms, underscores the need to standardize and simplify these complex concepts.

We found 42 studies that addressed plasticity as a central hypothesis and 20 that mentioned it descriptively, confirming its importance in Porifera research. While its inclusion in reviews indicates broad recognition, nearly one‐third of the studies treat plasticity as purely descriptive without exploring its functional or adaptive roles. This gap highlights a significant opportunity for further research into its underlying significance.

Among the screened articles, morphological responses were the most frequent (33 records), indicating that changes in form and structure are the most widely documented types of sponge plasticity. Physiological (17 records), molecular (7 records), and developmental (2 records) responses were also observed, although to a lesser extent. These findings reflect the complexity of phenotypic adaptations and the variability of levels at which phenotypic plasticity can occur.

Interestingly, cellular plasticity was well‐represented among the recorded plastic responses. Although we have not classified cellular plasticity as an exclusive category, its presence across all categories (morphological, physiological, molecular, and developmental) suggests that plastic responses are interconnected rather than compartmentalized. This reflects the integrated nature of biological systems, a key debate in phenotypic plasticity research. While plasticity involves interacting response levels, the prevalence of morphological and cellular data may indicate a study bias or simply the greater ease of measuring these specific traits in sponges.

The most frequent environmental triggers for plasticity were hydrodynamics (9), temperature (7), depth (6), and irradiation (5). Less common factors included seasonality (4), substrate (3), symbiosis (3), predation (3), sedimentation (2), pH (2), and tides (1). Notably, 22 of the 43 analyzed publications provided no explanation for environmental triggers. Every study identifying morphological plasticity cited hydrodynamics as the primary cause, reflecting the critical role of water movement, light, and turbidity in determining sponge shape (Schönberg 2021).

Research on sponge phenotypic plasticity is heavily biased toward Demospongiae (62 of 73 identified species), with few studies on Calcarea (8) or Homoscleromorpha (3), and none on Hexactinellida (Table 2). This taxonomic bias likely reflects Demospongiae's status as the most diverse class (Van Soest et al. 2012), yet it means a general phylum characteristic is being studied through only one group. After all, do all classes of Porifera exhibit such remarkable phenotypic plasticity, or did we attribute a feature of one of its classes to an entire phylum? Insufficient empirical data on other classes hinder a phylum‐wide understanding of plasticity. Broader studies across all Porifera classes are necessary to avoid drawing conclusions from limited data.

Most studies in our review focused on adults (70 species), with only a few examining gemmules (1 species), embryos (1 species), and primmorphs (4 species) (Table 2). While studying early stages is challenging (Lanna et al. 2024), these phases are crucial for understanding the developmental mechanisms and genotype‐phenotype connections underlying plasticity (Charrier et al. 2012). Research shows that early environmental cues can shift developmental pathways (Palumbi 1986; Müller and Müller 2003), with adults responding through morphological, cellular, and gene expression changes (Koziol et al. 1998; Pulido Mantas et al. 2024; Lavrov and Kosevich 2016). The scarcity of early‐stage research leaves a significant gap in our knowledge of how sponge plasticity originates and is regulated throughout life.

Consistent with general literature, sponge plasticity occurs across all organizational levels (see, e.g., Gaino et al. 1995). We categorized these “plasticity approaches” into population (22 articles), individual (13), and intraindividual/cellular (7) levels. This classification distinguishes between studies focusing on single‐genotype responses, population‐wide environmental variability, and internal cellular differentiation. The emphasis on population‐level plasticity in the sponge literature is likely driven by the specific research fields and methodologies employed (e.g., taxonomy and morphology). In these studies, plasticity is commonly used to explain group‐level adaptive responses to different environments (Bavestrello and Sarà 1992; Pronzato et al. 1998; Meroz‐Fine et al. 2005; López‐Legentil et al. 2010; Andreakis et al. 2012; Guardiola et al. 2016; Shaffer et al. 2018; Vicente et al. 2019; Idan et al. 2020).

These findings raise two theoretical questions that Forsman (2015) previously addressed. First, can applying a single concept to diverse context‐dependent variations be justified by a unified theoretical framework? Second, given the rich terminology, when is it justified to use distinct concepts? Clarifying these interpretations remains a work in progress, particularly in sponge research. The following sections explore these questions by analyzing plasticity‐related terms in sponge literature.

### 3.2 Sponge Cellular Plasticity

Metazoans comprise multiple cell types that arise from an isogenic genome (Qin and Tape 2024). To achieve such heterocellularity, cells undergo several differentiation processes (i.e., the inherent capacity of stem or progenitor cells to change into distinct specialized cell types). However, some cell‐type transitions require a certain degree of cellular ‘flexibility’ to alter cell fate in response to external changes and physiological processes. These cellular transitions can occur through a noncanonical differentiation process termed "cellular plasticity" (Qin and Tape 2024).

A hallmark of sponge biology is the presence of totipotent/pluripotent stem cells that confer significant anatomical and tissue plasticity throughout their life cycle (Funayama 2018; Ereskovsky et al. 2021). Sponges possess a unique capacity for cellular transdifferentiation, where differentiated cells can move, switch functions, and adopt new phenotypes as needed (Ereskovsky et al. 2021). This flexible differentiation system underpins a continuous remodeling process known as "chronic morphogenesis" Mi, which contributes to high regeneration capacity, growth, and somatic tissue reconstruction during sexual and asexual reproduction (Gaino and Burlando 1990; Gaino et al. 1995; Ereskovsky et al. 2021).

Sponge stem cells include two types with varying degrees of potency: (i) archaeocytes and equivalent ameboid cells, cells in the mesohyl capable of differentiating into all other cells (totipotent cells); and (ii) choanocytes, flagellated cells that constitute the choanocyte chambers which are capable of transdifferentiating into some other sponges’ cells (Funayama 2013; Lanna et al. 2024; Ereskovsky et al. 2024). Cellular complexity varies across sponge classes, with the potency of archaeocytes and choanocytes differing among groups (Funayama 2018). For instance, sponges lack a specific germline; somatic stem cells instead transdifferentiate into germ cells to initiate gametogenesis (Funayama 2018; Lanna et al. 2024). In demosponges, archaeocytes typically form oocytes and choanocytes originate from sperm, whereas in hexactinellids, oocytes derive from archaeocyte‐like cells. In calcareous and homoscleromorph sponges, both gametes derive from choanocytes. Thus, the primary stem cell type depends on phylogenetic position and life stage (Funayama 2018).

Sponge stem cells underpin the cellular plasticity and regenerative capacity of the phylum. In the literature, there is no clear distinction between these concepts regarding sponges. Cellular plasticity is heavily emphasized, often referring to totipotency/pluripotency, cellular movement, tissue rearrangement, regeneration, and physiological changes during reproduction (Gaino et al. 1995; Müller and Müller 2003; Meroz‐Fine et al. 2005; Gilis et al. 2011; Fassini et al. 2012; Ereskovsky et al. 2013b; Lavrov and Kosevich 2016; Finoshin et al. 2020; Kravchuk et al. 2023). Beyond sponges, cell potency, plasticity, and regeneration represent distinct yet related processes. But how do these phenomena relate?

The link between plasticity and potency in sponge stem cells often blurs their conceptual boundaries. Here, we defend that cellular differentiation (and potency), cellular plasticity, and regeneration are different but related processes. Cellular plasticity is defined by the cell's innate capacity to undergo phenotypic modifications in response to external stimuli without altering its genotypes (Tata et al. 2021; Qin and Tape 2024). This differs from the cell potency concept, which refers to the inherent capacity of stem or progenitor cells to differentiate based on a genetic program, as observed during development and tissue regeneration (Riveiro and Brickman 2020). Regeneration involves the coordinated activation of these processes to restore lost or damaged tissues. Both potency and plasticity require cellular reprogramming triggered by environmental shifts or genomic manipulation (Smith et al. 2016). Driven by stem cells, cellular plasticity enables sponges to adapt through lifelong differentiation and transdifferentiation, processes essential for regeneration and homeostasis. While molecular mechanisms remain poorly understood, investigating the sponge genetic toolkit could illuminate the evolutionary origins of metazoan stem cells.

Qin and Tape (2024) outlined a stepwise approach to studying cellular plasticity: (1) defining the concept, (2) developing cellular models, (3) applying single‐cell technologies, and (4) using lineage tracing to map dynamic behaviors over time. A sponge model system should replicate the entire sequence of plasticity‐driven transitions, from initial to final states, including all intermediates. Second, the model should enable precise, noninvasive monitoring in space and time. Finally, it must allow systematic perturbations for functional analyses of specific components. Currently, a defined sponge model for investigating plasticity is lacking; however, the pursuit of understanding cell fates has driven extensive research. Over recent decades, the following species have uniquely advanced our knowledge of sponge plasticity, lineages, and molecular mechanisms.

As the first sponge with a sequenced genome, *Amphimedon queenslandica* provides a foundation for studying the evolution of gene regulatory networks (Srivastava et al. 2010). Recent transcriptomic analyses of this species have resolved cell‐specific transcriptional landscapes, uncovering fate trajectories and transitional states that underpin cellular plasticity (Conaco et al. 2012; Fernandez‐Valverde et al. 2015; Wong et al. 2019). Previous works on *Geodia cydonium* identified extracellular matrix (ECM) remodeling as a key driver of cellular transitions, particularly during archaeocyte differentiation and dedifferentiation (Koziol et al. 1998). Similarly, *Suberites domuncula* became a prominent model through *in vitro* cultures, which allowed researchers to observe how archaeocytes differentiate into various cell types in response to external signals like retinoic acid and cytokines (Koziol et al. 1998; Custodio et al. 1998; Wang et al. 2013). The freshwater sponge *Ephydatia fluviatilis* offers a dynamic view of transdifferentiation (Funayama et al. 2010). Its ability to be cultivated from gemmules enables synchronized regeneration assays, revealing that “terminally differentiated” choanocytes can dedifferentiate into archaeocyte‐like states (Funayama et al. 2005). Finally, *Ephydatia muelleri* is a promising model due to its recently sequenced genome (Kenny et al. 2020) and well‐documented physiology (Leys et al. 2025).

Studies on *Sycon ciliatum* have advanced our understanding of cell lineage differentiation and regeneration in the class Calcarea. Its regenerative capacity mimics postlarval development, offering insights into sponge cell plasticity (Soubigou et al. 2020). Key findings include the rapid activation of developmental signaling pathways during wound healing and the role of long non‐coding RNAs (lncRNAs) in regulating cell fate and tissue regeneration (Gaiti et al. 2018; Caglar et al. 2021). These mechanisms highlight how *S. ciliatum* coordinates dynamic cellular responses to injury and maintains identity during regeneration (and also in *A. queenslandica*; Gaiti et al. 2018).

*Oscarella lobularis* (Homoscleromorpha) has become a key model for studying cellular plasticity, epithelial morphogenesis, and metaplasia (Ereskovsky et al. 2009, 2015). During regeneration, its differentiated cells undergo dedifferentiation and reprogramming to repair tissue and maintain cell lineages. This species exhibits significant epithelial reorganization during wound healing, allowing researchers to observe how cells alter their identity and function in response to injury (Renard et al. 2021). Recently, the ability to produce and maintain thousands of clonal buds *in vitro* has opened new avenues for controlled molecular and cellular experiments (Rocher et al. 2024).

This list could be extended, as many studies on regeneration and transdifferentiation address phenotypic plasticity without using the term. Despite ongoing research, the lack of an integrated model prevents researchers from combining these findings. This gap is exacerbated by studies that isolate specific cellular processes rather than considering the full spectrum of plasticity. Furthermore, as mechanisms likely differ across classes, the absence of comprehensive, cross‐class models prevents addressing sponge cellular plasticity as a cohesive concept.

### 3.3 Sponge Morphological Plasticity and the Environmental Influence

Morphology has long been a major topic of interest among sponge biologists; yet it remains perhaps the most complex facet of sponge phenotypic plasticity to interpret. The widespread view that sponges are highly plastic stems from the diverse forms their bodies can assume. Distinguishing whether these morphological variations result from true plastic responses, genetic differences, selective survival, or developmental noise remains a significant challenge. In this session, we will delve into these questions. As a definition, “sponge morphology” here refers to any feature included in internal morphology (tissue organization, composition, number of cells, spongin skeleton, and spicules) and external morphology (color, shape, mass, branches, texture, form of growth, oscula).

Sponges’ body shapes can range from massive spheres to encrusting or arborescent forms, tall branches, thin volcano‐like mounds, and more, depending on the species and the environment in which the organism is found. Despite this diversity, high intraspecific variation, often environmentally influenced, complicates feature comparison and species delimitation (Schönberg 2021; Cavalcanti et al. 2007; Wulff 2023). Taxonomic studies often cite phenotypic plasticity to explain individual differences, even without evidence that such variations are adaptive or consistent (López‐Legentil et al. 2010; Guardiola et al. 2016; Lopes et al. 2018). This ambiguity blurs species boundaries and undermines morphological traits as diagnostic tools (Pansini and Pronzato 1990). The resulting lack of species‐specific external features necessitates the use of molecular markers to refine sponge taxonomy.

It is difficult to determine exactly how much influence the genotype and environment have on sponge morphology. We usually attribute morphological differences among animals to changes in the spatiotemporal expression of genes conserved across disparate species (Colgren and Nichols 2020; reviewed in Carroll 2008). Morphological determinants are understood primarily through bilaterian models (e.g., *Drosophila*, *Caenorhabditis*, *Xenopus*, *Strongylocentrotus*, *Danio*, and *Mus*), leaving significant gaps in early‐diverging lineages (Colgren and Nichols 2020). The biology of ctenophores, placozoans, cnidarians, and sponges is so divergent from these classical models that it challenges our understanding of animal patterning evolution. The contrasting roles of patterning and plasticity in determining sponge morphology exemplify this challenge. The sponge body form is primarily driven by the functional necessity of efficient water processing for feeding and excretion (Bond and Harris 1988; Leys and Hill 2012). This requirement triggers constant adjustments that are highly dependent on environmental conditions (Colgren and Nichols 2020; Ereskovsky et al. 2021).

The aquatic environment's interacting abiotic and biotic factors create diverse microhabitats, leading to expected environment‐phenotype matching (Schönberg 2021). As filter feeders, the sponge body shape is highly influenced by hydrodynamics, sedimentation, substrate type, and biological interactions (Bell and Barnes 2000). Ecological studies frequently focus on the adaptive functions of sponge morphology, explaining the literature's emphasis on this response (Bell and Barnes 2000; Wulff 2006; Schönberg 2021). Despite being well‐investigated, sponge morphology is often linked to broad conditions rather than specific factors, necessitating further discussion on their biological and taxonomic impacts.

Among the environmental factors impacting sponge morphology, we found that hydrodynamics (flow structure and strength) is the most influential (20% of the studies). In high‐current environments, sponges develop massive or irregular shapes to minimize drag and maximize nutrient uptake. At high‐energy sites, encrusting morphologies dominate because their high basal area‐to‐volume ratio prevents displacement (Wilkinson and Vacelet 1979; Pronzato et al. 1998; Bell and Barnes 2000; Hill and Hill 2002; Gökalp et al. 2020; Schönberg 2021). In contrast, sponges in low‐current areas may adopt pedunculate, papillate, or arborescent morphotypes to prevent sediment accumulation (Wilkinson and Vacelet 1979; Pronzato et al. 1998; Bell and Barnes 2000; Gökalp et al. 2020; Schönberg 2021). These structural features optimize food acquisition and stability under specific hydrodynamic conditions. However, notably, this variation is often observed across communities rather than within individual species.

Efforts to understand how sponge growth and genetic expression navigate the interplay between a relatively simple bauplan and intense environmental influences are not new. For decades, research has demonstrated that the physical environment plays a fundamental role in constructing the sponge body, whether through the availability of essential materials incorporated during development or by hydrodynamically guiding the architecture of the aquiferous system. This long‐standing quest drove the development and application of mathematical models to simulate the interactions between genetic regulation and physical forces. By combining these morphological simulations with experimental investments, it has become possible to bridge different biological scales. For instance, this integrated approach connects molecular mechanisms, such as how high silicate concentrations upregulate the silicatein gene to activate local spicule production and skeletal deposition, with a regulatory network model that controls the spatio‐temporal separation of skeletogenesis and aquiferous system formation (e.g., Kaandorp 1991; Kaandorp and de Kluijver 1992; Kaandorp et al. 2008). This mechanical link between environment and morphogenesis provides a prime example of how we can begin to integrate distinct levels of biological organization, setting the stage for empirical studies aimed at quantifying environmentally driven morphological variation in natural systems.

Following the literature reviewed by Wulff (2023), transplantation to environments with stronger water motion leads to thicker spicules in *Halichondria panicea* (Palumbi 1986) and *Cinachyrella australiensis* (Mcdonald et al. 2002) and increased spicule density in *Tetilla* sp. (Meroz‐Fine et al. 2005). Similarly, *Petrosia ficiformis* and *Petrosia (Petrosia) clavata* develop more robust spicules under intense water flow (Bavestrello and Sarà 1992). Depth also influences reproduction: shallow‐water *Tetilla* sp. produces smaller, denser oocytes and releases gametes earlier than deep‐water populations (Meroz‐Fine et al. 2005). Additionally, transplanting the *Haliclona caerulea/Jania* association to shallower depths results in smaller, denser oscula, narrower spicules, and increased attachment area and organic content (Carballo et al. 2006).

Despite the emphasis on hydrodynamics, most studies consider multiple interconnected factors. For instance, *Anthosigmella varians* exhibits plasticity in spicule density in response to predation, whereas *forma incrustans* fails to branch in predator‐free, low‐energy environments (Hill and Hill 2002). This suggests that while some traits are plastic, others may remain fixed depending on the species and context. In line with this multifactorial perspective, the previously cited article by Carballo et al. (2006) explored plasticity in the mutualistic association between the sponge *Haliclona caerulea* and the red alga *Jania adherens*. This relationship, in which the alga provides structural support and the sponge offers protection, significantly influences morphology in response to external factors. Depth affected tissue size and density, while hydrodynamics drove the formation of resistant morphologies under strong currents. Additionally, solar irradiation indirectly promoted sponge development by enhancing algal growth. These interactions exemplify the difficulty in isolating specific environmental drivers of sponge morphological change.

Beyond hydrodynamics, ecology, and light, factors like sedimentation (turbidity, deposition, and abrasion) also influence sponge morphology and plasticity. As suspension feeders, sponges face limited filtration efficiency and potential tissue necrosis or death from high sediment levels. The classic study by Pronzato et al. (1998) demonstrated that sedimentation influences *Spongia* morphology and physiology. Sponges in high‐sedimentation habitats developed compact shapes, smooth surfaces, and larger oscula to minimize accumulation and improve filtration. While these adaptations prevent clogging, excessive sedimentation still reduces overall efficiency, limiting growth and survival. Temperature variations affect metabolic rates and tissue composition, influencing growth and resilience. For instance, *Chondrilla* aff. *nucula* in Brazil exhibits seasonal changes in spicule and cell characteristics (Cavalcanti et al. 2007). Between 2017 and 2023, marine heatwaves significantly altered the volume and biomass of *Sarcotragus foetidus*, highlighting thermal stress as a driver of morphological plasticity (Pulido Mantas et al. 2024). Additionally, Antarctic demosponges show metabolic plasticity through seasonal shifts in oxygen consumption and nutrient excretion, allowing them to adapt to temperature and ice fluctuations (Morley et al. 2016). Light availability (irradiation, luminosity, and UV exposure) significantly influences sponge morphology and physiology. Sponges often adjust their shapes to optimize surface area for photosynthetic symbionts: those in high‐light areas typically adopt flat or encrusting forms to maximize light capture, while those in shaded environments develop different shapes to reduce exposure (Wilkinson and Vacelet 1979; Meroz‐Fine et al. 2005; Wulff 2023). Furthermore, light levels influence bacterial community composition, resulting in distinct microbial associations between illuminated and low‐light habitats (Cleary et al. 2024).

Our review shows that research predominantly focuses on population‐level responses, often utilizing non‐clonal techniques to examine group‐wide adaptations (see, for instance, Miller et al. 2001; López‐Legentil et al. 2010; Gilis et al. 2011; Andreakis et al. 2012; Guardiola et al. 2016; Galitz et al. 2018; Shaffer et al. 2018; Idan et al. 2020). However, the field lacks a standardized definition of phenotypic plasticity, with interpretations varying across research approaches. To study environmental influence on sponges, researchers can use experimental transposition, testing phenotypic plasticity by observing how replicates (ideally clones) respond to different environments (Schlichting 1986). The experimental results are "reaction norms", i.e., the range of phenotypic variation produced by a single genotype across different environments (Stearns 1992; Schlichting and Pigliucci 1998; David et al. 2004). In sponges, these studies can be categorized into two groups, i.e., "non‐clonal", where entire organisms or fragments are transplanted without being treated as genetic replicates (Meroz‐Fine et al. 2005), and "clonal", where fragments are transplanted as genetic replicates (Wilkinson and Vacelet 1979; Hill and Hill 2002; Trussell et al. 2006; Carballo et al. 2006; Slattery et al. 2016). The clonal approach is preferred for identifying genotype‐specific plasticity. This approach is feasible because sponges’ regenerative capacity and asexual reproduction, via fragmentation, budding, or gemmulation, produce genetically identical offspring (Lanna et al. 2024). While genotypic control is often difficult in animal studies, clonal sponges allow direct assessment of adaptive traits (Todd 2008; Wulff 2023). Consequently, the ease of obtaining clones makes sponges excellent models for manipulative experiments, but this area of research remains open for further exploration.

It remains uncertain whether internal and external morphological variations in sponges are: (1) driven by natural selection in response to environmental conditions, where only phenotypes that are adapted to survive persist in that habitat, or (2) if they represent a morphological adaptation (plasticity) of species/genotypes to these fluctuating conditions, or (3) possibly both scenarios working together. The first scenario suggests that evolutionary pressures, such as competition and predation, favor traits that enhance survival. Over time, natural selection preserves these advantageous phenotypes, ensuring that the fittest genotypes predominate. In contrast, the second scenario proposes that sponges adjust their morphology in real‐time to immediate environmental fluctuations. These adjustments result from the organism's capacity to alter form and function without genetic change. Alternatively, natural selection and phenotypic plasticity may act simultaneously; immediate morphological adjustments could lead to genetic canalization if specific plastic traits confer survival advantages. Thus, sponge variation likely reflects both immediate, reversible responses and long‐term, genetically fixed adaptations.

We cannot know which scenario best explains each of the previously discussed morphological and physiological changes. Consequently, determining trait adaptivity requires caution, as adaptive plasticity must be shaped by natural selection (Dewitt and Scheiner 2004). Wulff (2023) first addressed this in sponges by integrating comparative and experimental data to distinguish *Aplysina fulva* and *Aplysina cauliformis*. These findings suggest phenotypic plasticity drives adaptation and diversification even among molecularly similar species, highlighting its role in ecological divergence.

Morphological plasticity in sponges remains unresolved, particularly whether variations stem from adaptive processes, genetic differences, or environmental factors. A multidisciplinary approach, combining genetic, ecological, and physiological analyses, is needed to disentangle these factors. Genetic studies identify trait heritability, while environmental assessments and transposition experiments isolate the roles of light, water flow, and nutrients. This integrative framework, alongside long‐term field studies, would clarify how plasticity manifests and the mechanisms driving sponge responses. Much remains to be understood regarding the adaptive and evolutionary role of phenotypic plasticity in sponges.

### 3.4 Rethinking Plasticity in Sponges and Final Considerations

As widely documented, sponges are remarkably plastic, with unique responses in external shape, cellular function, and regeneration that are fundamental to their survival (Bergquist 1978; Gaino et al. 1995). However, this plasticity is not infinite; it is constrained by species‐specific limits, environmental conditions, and biological traits.

Sponge plasticity is biased by biological and energetic costs, as remodeling can compromise survival and reproduction (Wulff 2006). For instance, optimizing morphology for filtration involves trade‐offs, such as reduced reproductive capacity or increased vulnerability to predators (Schönberg 2021). Maintaining this flexibility is energetically demanding, and any impairment to water flow or skeletal integrity ultimately reduces competitive fitness. For example, Meroz‐Fine et al. (2005) showed that *Tetilla* sp. balances reproductive investment against environmental stability; shallow‐water populations release gametes earlier, while deep‐water populations invest more in larger oocytes. This illustrates how sponges allocate energy between reproduction and other demands, like spicule production, based on environmental challenges.

These biases also involve the sponge *bauplan*. While plasticity allows for adjustment, developmental complexity and structural interdependencies bias the extent of change (West‐Eberhard 2003). Plastic phenotypes are biased by the need to maintain an efficient water flow and filtration system; significant deviations could compromise survival (Leys and Hill 2012). Additionally, historical contingency and evolutionary history limit the direction of change (Gould 2002). For example, a sponge lacking a spicular skeleton, such as *Oscarella*, cannot produce spicules even when exposed to high hydrodynamic stress.

Despite these biases, sponges’ exceptional plasticity makes them ideal for studying the mechanisms and limits of this phenomenon. Whether this plastic capacity is an ancestral state or a derived adaptation remains unknown (Colgren and Nichols 2020), necessitating a focused research agenda. Nonetheless, sponges are key models for exploring the evolution and ecological significance of plasticity across metazoans.

As biologists, we try to understand life through the patterns and principles that govern complex biological phenomena. But biology is often unpredictable. Discussing evolutionary concepts is challenging due to the complexity of biological processes, diverse interpretations, and the dynamic nature of the field. Many such concepts are abstract, interdisciplinary, and frequently revised, requiring an integrated approach. That is the case of phenotypic plasticity.

In sponges, phenotypic plasticity can be explored through multiple conceptual lenses. Rather than judging the "correctness" of specific terminology, we must question the motives behind its use. Sponge plasticity requires a standardized framework to classify responses across biological scales. This uniform approach distinguishes plasticity from related phenomena, ensuring conceptual precision and consistency.

Within our study, we have identified different approaches to refer to phenotypic plasticity in sponges, which we classified into: (1) intraindividual/cellular plasticity, (2) individual plasticity, and (3) population plasticity. The intraindividual/cellular approach demonstrates how a single organism can adjust its cellular structures and/or functions in response to the environment. The individual category examines how an entire organism can alter its morphology, physiology, or behavior to adapt to varying conditions. And finally, the population approach reveals how different individuals of the same species can vary in their responses to the environment. This classification distinguishes between single‐organism changes and collective population patterns. From an evolutionary perspective, it helps elucidate which mechanisms at different biological scales are subject to evolutionary processes. Adopting this uniform approach facilitates comparative studies, refines developmental and ecological definitions, and provides a framework for evolutionary comparisons.

In light of these findings, this review concludes that the indiscriminate use of "phenotypic plasticity" and the neglect of genetic variability have created a conceptual confusion that masks the true evolutionary mechanisms within the phylum Porifera. While environmental factors clearly shape sponge morphology, the functional role of these changes remains poorly understood, requiring a shift in focus toward early life stages and the distinction between real‐time plastic responses and natural selection. To bridge these gaps, we propose a standardized framework, categorized into intraindividual, individual, and population levels, to clarify biological constraints and provide the terminological precision necessary for future molecular and ecological research.

## Data Availability

Data sharing is not applicable to this article as no datasets were generated or analyzed during the current study.
